# Congenital Hypothyroidism in Preterm Newborns – The Challenges of Diagnostics and Treatment: A Review

**DOI:** 10.3389/fendo.2022.860862

**Published:** 2022-03-18

**Authors:** Martyna Klosinska, Agnieszka Kaczynska, Iwona Ben-Skowronek

**Affiliations:** Department of Pediatric Endocrinology and Diabetology, Medical University of Lublin, Lubin, Poland

**Keywords:** congenital hypothyroidism, hypothyroxinemia, neonatal screening, preterm newborns, thyroid hormones

## Abstract

Preterm newborns are forced to adapt to harsh extrauterine conditions and endure numerous adversities despite their incomplete growth and maturity. The inadequate thyroid hormones secretion as well as the impaired regulation of hypothalamus-pituitary-thyroid axis may lead to hypothyroxinemia. Two first weeks after birth are pivotal for brain neurons development, synaptogenesis and gliogenesis. The decreased level of thyroxine regardless of cause may lead to delayed mental development. Congenital hypothyroidism (CH) is a disorder highly prevalent in premature neonates and it originates from maternal factors, perinatal and labor complications, genetic abnormalities, thyroid malformations as well as side effects of medications and therapeutic actions. Because of that, the prevention is not fully attainable. CH manifests clinically in a few distinctive forms: primary, permanent or transient, and secondary. Their etiologies and implications bear little resemblance. Therefore, the exact diagnosis and differentiation between the subtypes of CH are crucial in order to plan an effective treatment. Hypothyroxinemia of prematurity indicates dynamic changes in thyroid hormone levels dependent on neonatal postmenstrual age, which directly affects patient’s maintenance and wellbeing. The basis of a successful treatment relies on an early and accurate diagnosis. Neonatal screening is a recommended method of detecting CH in preterm newborns. The preferred approach involves testing serum TSH and fT4 concentrations and assessing their levels according to the cut-off values. The possible benefits also include the evaluation of CH subtype. Nevertheless, the reference range of thyroid hormones varies all around the world and impedes the introduction of universal testing recommendations. Unification of the methodology in neonatal screening would be advantageous for prevention and management of CH. Current guidelines recommend levothyroxine treatment of CH in preterm infants only when the diagnose is confirmed. Moreover, they underline the importance of the re-evaluation among preterm born infants due to the frequency of transient forms of hypothyroidism. However, results from multiple clinical trials are mixed and depend on the newborn’s gestational age at birth. Some benefits of treatment are seen especially in the preterm infants born <29 weeks’ gestation. The discrepancies among trials and guidelines create an urgent need to conduct more large sample size studies that could provide further analyses and consensus. This review summarizes the current state of knowledge on congenital hypothyroidism in preterm infants. We discuss screening and treatment options and demonstrate present challenges and controversies.

## Introduction

**Figure 1:**
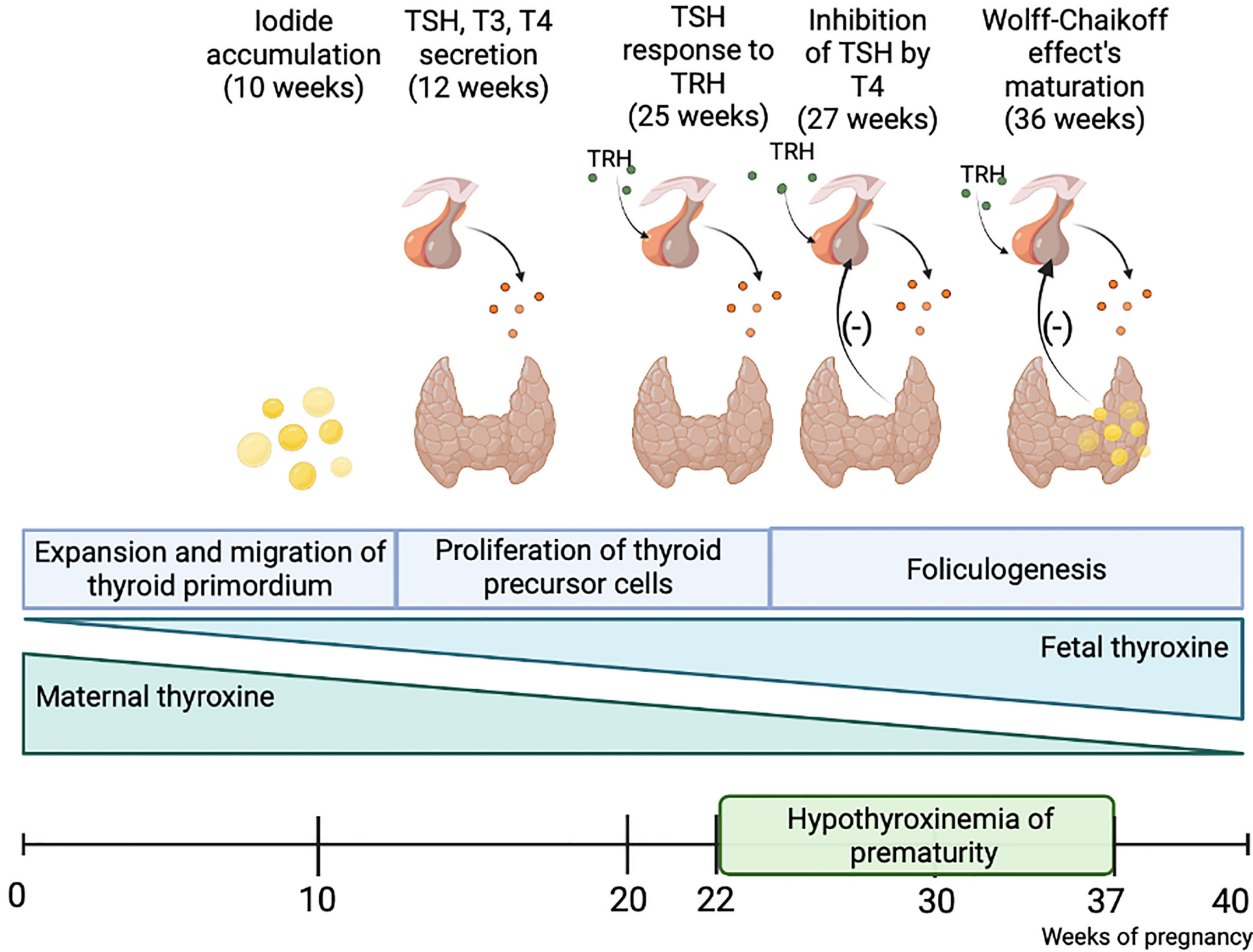
Fetal thyroid gland maturation. The fetus is dependent on maternal thyroid hormones supplementation until the end of the first trimester. The hypothalamus-pituitary-thyroid axis is not sufficiently developed until the end of pregnancy, thus preterm-born children present a disorder called hypothyroxinemia of prematurity, which results in numerous consequences. Created with BioRender.com.

Thyroid hormones (TH) play a significant role in the development of every neonatal organ, especially brain. Insufficient maternal TH levels during the first trimester of pregnancy are associated with numerous disfunctions noticeable before, right after birth and later in an adult life (1). However, it is commonly known, that fetus is dependent on the maternal TH supply only until the end of the first trimester (2, 3). In the eighth week of gestation fetal hypothalamus, as well as fetal gut and pancreas in a lesser degree, begin to produce the thyrotropin-release hormone (TRH), which stimulates a pituitary gland to secrete the thyroid-stimulating hormone (TSH). In the tenth week fetal thyroid starts to accumulate iodine, produce thyroglobulin, and express TSH receptors. Simultaneously, fetal thyroxine-binding globulin (TGB), TSH and T4 levels begin to increase and double up until the term. Serum total T4 concentrations reach a value of 130 nmol/L with plateau at 35-37 weeks (4). The hypothalamic-pituitary-thyroid axis starts to mature by the second trimester of gestation. According to the new consensus by the European Society for Pediatric Endocrinology and European Society for Endocrinology, congenital hypothyroidism is defined as the hypothalamic-pituitary-thyroid (HPT) axis’s insufficient development, which is particularly observed in premature newborns, and results in numerous complications including an impaired thyroid activity and inadequate TH secretion (**Figure 1**) (5).

Statistic data suggests that globally 10,6% of births occur before 37 weeks’ gestation, which leads to about 15 million preterm births every year. Four percent of them occur before completed 28 weeks of gestation, while moderate to late preterm births (at 32-36 completed weeks) stand for 84% (6). Although many factors have been proven to increase the risk of spontaneous preterm birth, the vast majority of them appear in women without a clear risk factor. However, there is strong evidence that elevated level of maternal TSH may result in pregnancy loss or preterm delivery (7–9). It indicates that thyroid hormones play a significant role in not only the development of fetal organism, but also have a huge impact on the pregnancy outcomes.

The prevalence of congenital hypothyroidism (CH) in general population is estimated between 1:2000 and 1:3000 newborns (10). Multiple screening programs confirm its higher incidence in preterm infants, with almost 50% occurrence (11–14). According to the data mentioned above, along with the week of gestation, the development of the hypothalamic-pituitary-thyroid axis accelerates. Because of that, clinical manifestations and the severity of thyroid disorders variate depending on the premature newborn’s gestational age (15). The main differences involve diverse thyroid hormonal levels, whose insufficiency induces neurodevelopmental deficits (16). The detrimental implications of these comorbidities contribute to the urgency of an effective treatment. With various possible therapeutic choices and their unanticipated outcomes, there is a great need for further research and analysis (17).

In this study our aim is to explore and analyze the foundations and indications of thyroid disorders in premature newborns, the treatment options, and possible challenges. The review consists of presentation and analysis of literature and previously published studies obtained from PubMed, Scopus, and Google Scholar databases.

## Causes of Thyroid Disorders in Preterm Neonates

The complexity of neonatal thyroid disorders is generally known and still to be thoroughly classified. Evaluated risk factors involve for instance advanced maternal age, medication during pregnancy, family history of thyroid disease, low birth weight, preterm birth, twin pregnancy or birth defects (18, 19). Due to the serious general condition of premature infants, there is a necessity for both a careful perinatal care and further clinical analysis (20).

### Maternal Causes of Thyroid Disorders

One of the most frequent bases of the newborn’s thyroid abnormalities is in the mother’s endocrine disorder. The association between isolated maternal hypothyroxinemia and preterm birth is currently observed (21). In 2019 Korevaar et al. published a meta-analysis concerning the hypo- or hyperthyroidism of mother correlating with infant’s thyroid function and preterm birth. After including 19 cohorts with a population of 47 045 pregnant women, it was stated that patients’ subclinical hypothyroidism, isolated hypothyroxinemia and TPO antibody positivity directly affect higher risk of preterm birth (22). Maternal subclinical hypothyroidism is also associated with the lower birth weight as well as newborns small for gestational age (SGA), which explains the need for a careful therapy during pregnancy (23, 24). However, in 2018 Varner et al. conducted multi-center randomized, double-masked, placebo-controlled thyroxine replacement trials in pregnancies in order to assess the probable neonatal benefits of maternal subclinical hypothyroidism/hypothyroxinemia treatment. The conclusions stated no statistically relevant difference in infants’ TSH levels, which questions the value of prebirth pharmacotherapy (25). Contrary, current guidelines emphasize the importance of treating maternal overt hypo- and hyperthyroidism both during pregnancy and before the conception. What is more, the recommendations favor implementing targeted screening in case of high-risk pregnancies rather than universal screening for thyroid disorders before or throughout the pregnancy (26).

Maternal thyroid disorders not only affect the duration of pregnancy and labor, but also directly influence the newborn’s neuropsychological development. Studies show that low maternal free thyroxine concentrations may impair infant’s psychomotor and cognitive abilities leading to underperforming in school, learning difficulties or even behavioral and emotional disorders (27). Authors specifically examine Graves’s disease in mothers’ association with thyroid disfunction and SGA in neonates (3, 28). As the disorder not only remains the main cause of hyperthyroidism in pregnant women, but also bears risk of severe consequences, it needs to be diagnosed and adequately treated as soon as possible (29).

### Pregnancy Complications

Preterm labors may occur as a result of placental abruption or insufficiency or other pregnancy-related complications, especially in hypo/hyperthyroid women (30, 31). Endangered pregnancies have been identified as a significant factor in preterm neonatal thyroid disorder. Pre-eclampsia carries a great risk of placental insufficiency, which may induce intrauterine hypothyroidism (32). It is also claimed that perinatal asphyxia results in lower TSH, T4, T3 and FT4 cord blood levels in newborns (33).

### Genetic Factors and Thyroid Malformations

Generally, thyroid disorders in preterm newborns occur spontaneously or because of previously mentioned risk factors. Nevertheless, plenty of studies yield information about familial hypothyroidism with prevalence up to 2% (34). The genetic etiology of the disease seems to explain extrathyroidal malformations reported in some cases of CH with numerous candidate genes to possibly be responsible (34–36).

Mentioned thyroid morphological defects are known as a separate risk factor for congenital hypothyroidism in preterm newborns (37). Total or hemi agenesis, ectopy and hypoplasia are objectively diagnosed in up to 85% cases of thyroid dysgenesis (36).

### Pre- and Postnatal Medications

Multiple medications implemented during pregnancy and in the postnatal stage have numerous diverse effects on infants. However, in most cases their usage is a necessity, thus possible consequences must be considered. Amiodarone, which is commonly used in pregnant women for treatment of maternal and fetal dysrhythmias, may cause infant’s hypothyroidism (38). The drug is rich in iodine and resembles thyroxine in structure, so its administration may alter thyroid function (39). In preterm newborns the impact of excess iodine from amiodarone on TH is linked to the disturbed Wolff-Chaikoff effect (40). In general, the oversupply of iodine inhibits not only its organification but also thyroglobulin proteolysis (41). Recent studies show that 18,2% of premature neonates administered with iodinated contrast media (ICM) had transient hypothyroidism (42, 43). Nevertheless, a trial by Rath et al. suggests that ICM may cause adverse thyroid effects only when its administration is not conducted carefully (44). Moreover, lower TH levels are observed in preterm breastfed infants after their lactating mothers had a CT scan with ICM (45). Bowden et al. described the inhibition of TSH release induced by glucocorticoids, somatostatin, and dopamine (46). However, Ekmen et al. suggests that TSH, T3 and T4 levels are not disturbed during dopamine infusions (47). Bearing in mind that dopamine is known to suppress thyrotropin release, there is an urgent need to conduct more trials concerning its impact on infant’s TH. Some studies emphasize the negative influence of glucocorticoids on TSH and thyroxine secretion. The trial by Shimokaze et al. suggests that very short-term glucocorticoid administration may cause marked changes in TH levels (48).

## The Effects of Thyroid Disorders in Preterm Infants

**Figure 2:**
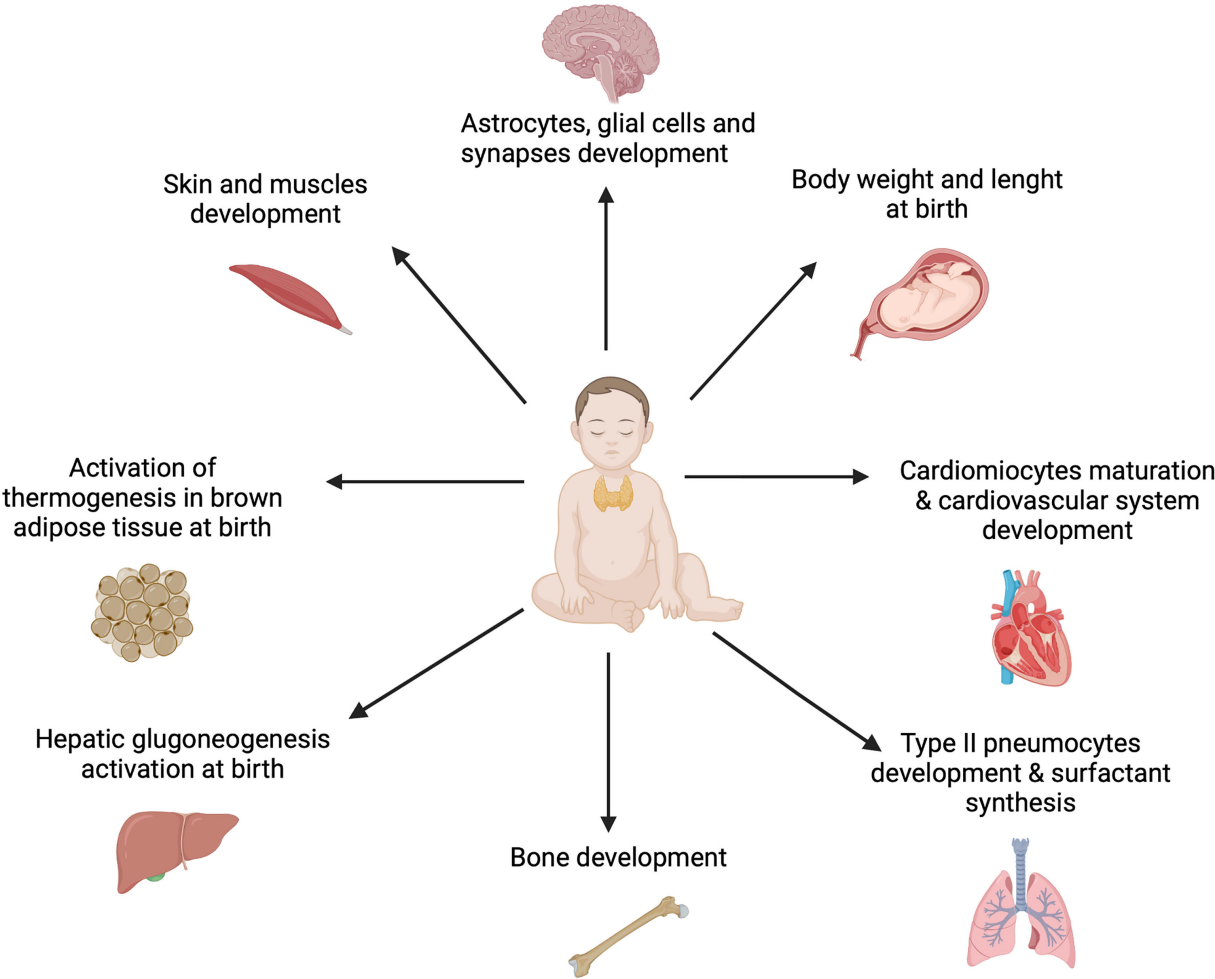
The role of thyroid hormones in fetal and infant development. Thyroid hormones are essential for the general accretion of fetal mass and to provoke developmental events in the fetal brain and somatic tissues from early in gestation. Moreover, they affect the production of other hormones and regulate tissue accretion near term. Furthermore, TH ensure activation of physiological processes as thermogenesis, gluconeogenesis, pulmonary gas exchange and cardiac adaptations at birth (49). Created with BioRender.com.

The thyroid gland produces triiodothyronine (T3) and thyroxine (T4) in response to pituitary gland stimulation (46). In cells T4 converts to T3 so that biofeedback mechanism maintains adequate levels of thyroxine for body metabolism and, in children, a proper growth and brain development. Thyroxine is a vital necessity for all the organs, tissues, and cells in the body to function normally. It also controls the body’s metabolic rate and other multiple processes (49) (**Figure 2**).

Thyroxine deficiency in early neonatal period may cause severe, irreversible mental and physical retardation, a condition known as cretinism. It is worth mentioning that fetal and newborn periods are the exact brain development stages when differentiation of numerous structures occurs in a short amount of time. Even small or subtle alterations in thyroid hormones levels may result in a disturbed brain development and a long-lasting or permanent deficits. As said before, fetus is dependent on maternal TH supply until the end of the first trimester (2, 3). Thus, during that period all the TH concentrations in fetal tissues are of maternal origin and even a small disruption in their transfer may result in brain development disturbances. Nevertheless, the fetus’s endocrine system begins to mature in early stages of gestation (50). On the other hand, CH is a type of TH deficiency which starts in late gestation and is caused by low TH fetus/infant production. Recently, multiple studies showed that TH have a profound role in oligodendrocytes and astrocytes’ maturation. For instance, T3 regulates cerebral cortex stratification, axon routing and cell migration by Cajal-Retzius and subplate cells (51). What is more, TH upregulate the differentiation and expression of neural cells as well as play a role in myelination and synaptogenesis (52, 53). De Souza Martins et al. demonstrated the positive influence of T3 supplementation on Myosin-Va (Myo-Va) expression. Myo-Va is a molecular motor protein that affects vesicle and RNA carriage. Its malfunction leads to abnormal axonal transport and synaptic function (54). Moreover, low TH levels affect glial cells. Opazo et al. presented that gestational hypothyroxinemia affects neural reactivity, causing its decrease in microglia and increase in astrocytes, while Flamant et al. suggested the link between a T3-dependent neurotropin secretion and oligodendrocyte progenitor differentiation (55–57). Furthermore, the synthesis of extracellular matrix proteins, which is under the TH control, has been linked to a reduced cortical thickness (57). Currently, the research concerning the role of TH in brain development gathers pace. However, the evidence is still limited, thus there is an urgent need to conduct more clinical trials.

### Epidemiology

Considering the remarkable progress and innovations in modern medicine, the survival rate of preterm newborns is constantly escalating (58). Although proper neonatal care should enhance neonates’ healthy development, in many cases it is insufficient (59). Consequently, there is an increasing incidence of congenital hypothyroidism (60, 61). Despite its estimated prevalence between 1:2000 and 1:4000 in infants, there is evidence about epidemiological differences dependent on the geographic location (62).

### Classification of Congenital Hypothyroidism in Preterm Newborns

Congenital hypothyroidism is a condition occurring in infants unable to produce a sufficient amount of thyroid hormone (thyroxine, T4), which is necessary for a normal metabolism, growth and brain development. The disorder develops sporadically and is rarely inherited.

Primary congenital hypothyroidism (PCH) is the most prevalent form of CH, because of which the serum T4 levels are low, whereas TSH levels are elevated (63). Moreover, it is not associated with neither prenatal nor risk factors. There are several types of primary CH, the most common form being caused by abnormal fetal development of the thyroid gland (36). Severe PCH entails the highest risk of neurodevelopmental impairment (64).

Permanent congenital hypothyroidism develops as a result of perpetual dyshormonogenesis or thyroid dysgenesis (65). Its risk factors are not fully analyzed yet (66). The prevalence in neonatal screenings is estimated at 1:748 (67). Surprisingly, it appears similarly in both pre- and term infants (68). As oppose to transient CH, it is said to occur more often in women than in men (69).

Transient congenital hypothyroidism (TCH) is more common in preterm neonates, with incidence of 1:1114 (67). The syndrome may develop as a result of maternal exposure to antithyroid medications or fetal exposure to maternal antithyroid antibodies (65, 70). What is more, the use of iodine-based skin disinfectants on premature infants can inhibit thyroxine production resulting in transient hypothyroidism (71). Untreated maternal hypothyroidism might lead to low fetal levels of thyroxine as well (71). TCH is characterized by decreased levels of thyroid hormones evaluated at birth which return to normal values after a few months or years of life (72).

Secondary or central congenital hypothyroidism (CHC) is observed in children after brain damage, intraventricular hemorrhage and in cases of an insufficiency of hypothalamus or/and pituitary gland (73). This disorder can only be diagnosed using screening tests detecting TSH and fT4 levels simultaneously or stepwise (74). As a matter of fact, CHC must be distinguished from T4-binding globulin deficiency (75).

### Hypothyroxinemia of Prematurity

In premature neonates, serum TSH, T4 and free T4 (fT4) concentrations tend to change dynamically according to their postmenstrual age (PMA). The initial high TSH values (with the peak at PMA=30 weeks) gradually decrease to reach the term infants’ level at PMA=38-40 weeks. Adequately, the T4 and fT4 levels are the lowest around PMA=26-27 weeks, only to also reach the norm at PMA=38-40 weeks (76, 77). Moreover, lower TGB concentrations are responsible for the decreased T4 serum levels.

Furthermore, some very low birth weight and extremely low birth weight premature neonates present delayed TSH elevation (dTSH), which is also known as “atypical hypothyroidism” (78–80). It occurs when second capillary screening at 1 month of age shows serum TSH concentrations higher than 20 mIU/L following a normal value below 15 mIU/L at first neonatal screening within first 4-6 days after birth (81). Woo et al. conducted a study to establish the prevalence of dTSH. The results identified the incidence of 1:58 in ELBW and 1:95 in VLBW infants. In comparison, it estimated 1:3029 in newborns with weight higher than 1500 grams, which proves dTSH dependence on low birth weight (82).

Transient hypothyroxinemia of prematurity is a term regarding the time of premature infant’s life with serum low thyroid hormone levels. The decreased values are suggested to originate from the immaturity of the hypothalamic-pituitary-thyroid axis (83). Studies mention various risk factors for this abnormality: lower gestational age, maternal pre-eclampsia, respiratory distress syndrome, mechanical ventilation, and dopamine infusions (32, 84, 85).

Although hypothyroxinemia of prematurity is a separate disorder, and not an additional form of congenital hypothyroidism, authors usually do not differentiate between them. Both conditions prevail because of preterm infants’ insufficient fetal thyroid evolution and multiple other risk factors described before (86). Clinical manifestations of the two diseases are very similar due to their strict origin from prematurity, thus it is almost impossible to classify them accordingly. Current guidelines do not specifically recommend treating hypothyroxinemia of prematurity with levothyroxine, unless TSH elevation is also observed (87). However, because of considerable difficulty in the exact diagnosis, it is usually treated as CH up to the necessary reevaluation after 6 months of life (5).

Iodine deficiency can be observed in preterm infants due to its insufficient amount in the parenteral nutrition and the rapid loss of maternal supply. It contributes to slow recovery from hypothyroxinemia of prematurity. Therefore, the newborn’s exposure to excess iodine, for instance iodine disinfectants or radiological contract infusions, leads to down-regulation of T4 and T3 levels, also known as Wolff-Chaikoff effect (71).

## Neonatal Screening in Group of Preterm Neonates

The early diagnosis of congenital hypothyroidism in preterm newborns is a pivotal factor conditioning the patient’s neuropsychological and motor development (88). Neonatal screening tests are most commonly performed by sampling blood from infant’s heel between the 2^nd^ and 5^th^ day after birth and evaluating the TSH, T4 and fT4 levels by using fluoroimmunoassay (89). The implemented TSH cut-off levels vary all over the world which provides different epidemiological reports and impedes the introduction of universal neonatal screening guidelines (90). For instance, in Thailand the optimal cord blood TSH value for recall is estimated at 30-40 mIU/L, while in Italy it stands at 10 mlU/L (91, 92). Kilberg et al. argue that TSH cut-off values in neonatal screening should be age-adjusted in order to detect mild cases of CH and persistent TSH elevations (93). The repetition of screening is said to be beneficial for the assessment of infant’s condition and treatment effects as well as classification of CH, permanent or transient, that allows an adequate patient’s management (94). Follow-up examinations are advised to be done after four, eight weeks and later every three months (90). Post screening strategies involving collecting of second blood specimen between 10^th^ and 14^th^ days after birth are recommended in neonates at risk of CH, which is preterm, low birthweight and sick infants. The mentioned newborns may present false-negative results in first neonatal screening (5).

The congenital hypothyroidism occurs when the detected TSH level is higher than the cut-off value and fT4 level is decreased. The pharmacotherapy with levothyroxine needs to be implemented. With tablet form the dose should be at 10-15 µg/kg/day and the medication should be taken 60 minutes before the breakfast (95, 96). In case of choosing the liquid form, the dosage should be the same, if not lower, as the suppression of TSH is higher due to higher absorption (97–99). Additionally, the liquid levothyroxine dissolves without an acid gastric pH, so it can be administered along with the meal (96). If TSH concentration is estimated within the range of the reference and cut-off values along with the decreased fT4 level, the diagnosis and treatment plan stand exactly as mentioned above. With normal TSH and fT4 values we can identify a proper thyroid function which does not require any medications. In case of decreasing of both TSH and fT4 concentrations, secondary CH is suspected and should be treated with levothyroxine at a dose 5-10 µg/kg/day or lower, at 1-2 µg/kg/day, in children with gland *in situ* or with isolated secondary CH (5, 90). In recently published Consensus Guidelines by an ENDO-European Reference Network Initiative Endorsed by the ESPE and ESE, if the serum TSH level estimates between 6-20 mU/l and fT4 concentration is within age specific references, or if the TSH value is higher than 20 mU/L and fT4 concentration is below the age-specific references, levothyroxine replacement therapy is immediately needed (5). Presented recommendations for dosages and values may differ regarding other guidelines, thus there is an emphasized need for unification of universal screening approaches.

## The Thyroid Hormone Replacement Therapy in Preterm Newborns

Described above numerous complications of congenital hypothyroidism indicate a tremendous need to conduct a therapy able to alleviate the course of the disease. Current guidelines recommend the supplementation of levothyroxine preparations (L-T4) in children with congenital hypothyroidism (5, 90). However, recent studies have yielded plenty of information on different outcomes of the thyroid hormone replacement therapy, both in preterm born children with transient hypothyroxinemia and CH (100–106). Indeed, here we describe current guidelines and the newest results of the clinical trials.

### Current Guidelines and Recommendations

Levothyroxine is known to be the most effective drug in congenital hypothyroidism. The crucial part of the treatment is the exact time of its initiation. Guidelines recommend starting the therapy not later than on the 14^th^ day of life. SGA, VLBW and ELBW newborns have greater risk of CH and its consequences, thus the time of the effective drugs action is short-up to four weeks after labor. Guidelines suggest maintaining the fT4 and fT3 levels not later than in 1-2 weeks. The initial dose of L-T4 depends on the severity of the disease. In primary CH levothyroxine in tablet form starts from 10–15 μg/kg/day and increases in children with severe CH (5). Oral administration on an empty stomach at least 30 minutes before eating is recommended (95, 96). Levothyroxine in liquid form can be administered along with the meal and the dosage is evaluated the same or lower than in the tablet form (96–99). The target dose should be adjusted according to the serum levels of TSH and fT4 to ensure stable euthyreosis. In suspected central CH levothyroxine dose is estimated at 5-10 µg/kg/day and in diagnosed CHC at 1-2 µg/kg/day. Reevaluations beyond the first 6 months of life are crucial to assess the need or its absence for further therapy (5). TSH level should be within the range of the reference values for age, and fT4 in the upper half of them. It is worth mentioning that inappropriate dose of levothyroxine (both insufficient and excessive) may cause multiple adverse effects and disturb treatment’s effects (107). The check-ups should be performed within specific time spans and accordingly to the patient’s needs (5, 90).

### Clinical Trials Do Not Correspond With Each Other

Some studies correspond with guidelines and show that preterm infants supplemented with levothyroxine perform significantly better in both cognitive and motor functions (100–102). However, as the research on that topic had been gathering pace, more studies not correlating with neither the previous ones nor the recommendations had emerged (104–106) (**Table 1**). In 2020 Ng et al. enrolled 153 infants before 28 weeks’ gestation to an explanatory double-blind, randomized, placebo-controlled trial. Children were supplemented with L-T4 or given the placebo until 32 weeks’ corrected gestational age. Neurodevelopmental outcomes after 42 months showed that the L-T4 supplemented group performed significantly better in motor, language, and cognitive function domains (100). Accordingly, in 2014 Noumra et al. showed that L-T4 supplementation prevents the developmental delay of extra low birth weight infants with transient hypothyroxinemia. Moreover, the trial proved the association of gestational age with serum levels of fT4 (101). The study by Suzumura et al. demonstrated that levothyroxine treatment in extremely preterm newborns initiated at the end of the first week of life could reduce the incidence of cerebral palsy (102). However, Van Wassenaer-Leemhuis et al. did not find any differences in mental or motor development and rates of cerebral palsy between the compared groups of infants born less than 28 gestational weeks, treated or not with levothyroxine (106). Van Wassenaer et al. reported there is no correlation between the initial plasma free thyroxine concentration and the effect of treatment. In the study 200 infants born before 30 weeks’ gestational age received orally L-T4 or placebo treatment for 6 weeks. The follow-up did not show any differences in mortality or morbidity between compared groups. In thyroxine-treated infants born before 27 weeks’ gestation the Mental Development Index measured at the age of 24 months was 18 points higher than in the placebo group, while among children born 27 weeks or later the same index was 10 points lower in the treated group than that of their counterparts (103). Hollanders et al. suggest no association between transient hypothyroxinemia of prematurity and neurodevelopmental outcome in young adulthood. The study was a part of 19 years follow-up project which included infants born very preterm and with very low birth weight. This long-time multicenter trial demonstrated no correlation of IQ score or motor function with hypothyroxinemia in preterm born children after adjustment for demographic and perinatal characteristics (105). Yoon et al. aimed to determine the incidence, etiology, and outcomes of the TSH elevation and its treatment in extremely low-birth-weight infants (ELBWIs). Indeed, the levothyroxine replacement therapy resulted in significantly higher TSH elevations, lower fT4 levels and significantly reduced mortality compared to untreated children. Nevertheless, according to the follow-up, the treatment had no significant effect on neurodevelopmental outcomes and growth (104).

**Table 1:** Summary of studies concerning the T4 treatment and its neurodevelopmental outcome.

Study	Intervention	GA	Total Group T4 vs placebo	Endpoint	Main results
**Clinical trials which correspond with current guidelines**					
Ng et al. (100)	T4, daily bolus, first 5 days iv, later orally; 8 µg/kg until 32 weeks’ corrected GA	<28 weeks	61 vs 57	Neurodevelopment at 42 months	Supplemented group performed significantly better in motor, language, and cognitive function
Nomura et al. (101)	T4, daily bolus; 5-10 µg/kg orally	— ELBW infants	18 vs 18	Neurodevelopment at 12 months corrected age	T4 prevents the developmental delay of ELBW infants
Suzumura et al. (102)	T4, daily bolus; 5-10 µg/kg for FT4 levels <0.8 ng/dL	<28 weeks	54 vs 60	Cerebral palsy at 3 years	Reduction of cerebral palsy incidence
Ben-Skowronek & Wisniowiecka (110)	T4, 5-10 µg/kg b.w./day since the second week of life	25-35 weeks LBW, VLBW ELBW	40 vs 52	Mental development in the 7th year of life	Improvement in long-term mental development
**Clinical trials which do not correspond with current guidelines**					
Vanhole, et al. (111)	T4, daily iv bolus, 20 µg/kg, d 1-14	<31 weeks	17 vs 17	Endocrine and clinical manifestations during first 2 weeks of life; Neurodevelopment at 7 months	No difference in clinical outcome and development
Van Wassenaer et al. (112)	T4, daily bolus, first 2-3 weeks iv, later orally; 8 µg/kg, d 1-42	<30 weeks	82 vs 75	Neurodevelopment at 24 months	No difference in total groups. Subgroup analyses: at 2 and 5yrs better outcome with T4, if Ga <27-29 weeks
Smith et al. (113)	T4, bolus, start iv: 10 mg/kg; then orally: 20 µg/kg, d 2-21	<32 weeks	29 vs 18	Chronic lung disease; Oxygen dependency at day 28	No effect on the incidence of chronic lung disease
Briet et al. (114)	T4, daily bolus, first 2-3 weeks iv, later orally; 8 µg/kg; d 1-42	<30 weeks	82 vs 75	Neurodevelopment at 24 months; Motor and neurologic outcome at 5.7 years	No difference in total groups. Subgroup analyses: at 2 and 5 years better outcome with T4, if Ga <27-29 weeks
Biswas et al. (115)	T3, continuous iv, 6 µg/kg/d + hydrocortisone 1 mg/kg/d; d 1-7	<30 weeks	125 vs 128	Death or ventilator dependence at day 7	No difference in adverse outcome
Van Wassenaer-Leemhuis et al. (106)	T4, daily bolus; 4-8 µg/kg iv or iodine 30 mg/kg iv; d 1-42	<28 weeks	14 (iodine) vs 62 (T4) vs 13	Cerebral palsy, mental and motor development at 3 years	No difference among groups
Yoon et al. (104)	T4, daily bolus; 10-15 µg/kg until the TSH normalized levels	>23 weeks and with ELBW	25 vs 22	Growth and neurodevelopment at 2 years	No difference among groups

GA, gestational age; ELBW, extremely low birth weight; T4, thyroxine; iv, intravenous; d, day.

Guidelines do not recommend the use of iodine in congenital hypothyroidism therapy (5, 90). However, some trials, regarding to iodine’s essential role in the synthesis of thyroid hormones, try to investigate whether its dietary supplementation affects thyroid function during the neonatal period. The meta-analysis performed by Walsh et al. did not show any effect of iodine intake on mortality or neurodevelopment in two-years follow-up. However, analyzed trials assessed the effect of prophylactic rather than therapeutic iodine supplementation (108). Further research conducted by Ares et al. revealed similar results. Ninety-four infants with very-low birth weight were enrolled to the trial and assigned into two groups. Children in the intervention group were treated everyday with iodine in oral drops, while the placebo group did not receive any supplements. Blood samples were collected for thyroid hormones and the neurodevelopment was assessed. The analysis showed a positive outcome on the blood levels of thyroid hormones. Infants in the supplemented group reached the recommended levels from the start of the trial. Nevertheless, positive neurodevelopmental effects of iodine intake were not found. The study suggest that preterm newborns are at high risk of iodine deficiency, thus their iodine intake should be monitored. Iodine supplementation should be considered if the intake is found to be insufficient (109).

## Conclusions

Despite considerable progress of management and treatment of congenital hypothyroidism, the disorder remains to induce substantial failures in infants’ neurodevelopment. An efficient solution that could influence not only a course of CH, but also its implications, is a proper establishment of how TH affects an infant’s brain, especially during pregnancy and early childhood. CH originates from multiple factors, thus their elimination could contribute to decreasing the prevalence of the disease. Unfortunately, there is still some uncertainty considering the effects of pre- and postnatal treatment of prematurity that could induce CH. In these cases, the avoidance of said risk factors seem almost impossible and require further evaluation and research. Moreover, the prognosis and possible therapeutic outcomes are crucially dependent on the early diagnosis of CH.

It is vital to identify the subtype of CH in preterm infants, as the exact classification enables an effective and appropriate management and treatment. Bearing in mind the frequency of transient CH, probable risk factors should be considered whereas necessary reevaluations and follow-ups ought to be implied in cases of uncertain diagnosis. We also need to be able to identify phenomena such as hypothyroxinemia of prematurity or delayed elevation of TSH, as their hormonal manifestations or implications substantially differ from typical CH forms.

Neonatal screening tests play a vital role in an effective disease recognition. Although a significant progress has been made in recent years, there is still a strong need for reevaluating and unifying screening guidelines to achieve coherent CH management and therapy. Authors specifically draw attention to mild cases of CH in which are sometimes impossible to detect. As mentioned before, reevaluations are crucial in cases of transient CH in order to assess the patient’s status and potential need or its absence for further therapy.

Current treatment guidelines recommend thyroid hormones substitution in children with congenital hypothyroidism. However, clinical trials have yielded plenty of information about diverse therapeutic results. Authors still aim to assess the most appropriate clinical approaches and dosages of levothyroxine. What is more, treatment models differ between studies and guidelines, thus comparing and analyzing their effects remains problematic. There are also questions considering the iodine supplementation. Although the guidelines do not recommend the use of iodine in the therapy of CH in preterm infants, it appears to be a subject of clinical trials and a possible addition to prevention. It is essential to conduct more research considering the therapy of CH in premature newborns as to unify the expected outcomes.

So far, medical society has gained plenty of up-to-date and thorough knowledge about congenital hypothyroidism in preterm infants. However, analysis of literature and current challenges presented in this review prove the urgent demand for further research.

## Author Contributions

MK contributed to the conception and design of the work, acquisited and analyzed of references for the work, and wrote the first draft the manuscript. AK contributed to the conception and design of the work, acquisited and analyzed of references for the work, and wrote the first draft the manuscript. IB-S contributed to the conception and design of the work, acquisited and analyzed of references for the work, and drafting the work or revising it critically for important intellectual content. All authors contributed to manuscript revision, read, and approved the submitted version the manuscript.

## Funding

Grant DS 415 from Medical University of Lublin.

## Conflict of Interest

The authors declare that the research was conducted in the absence of any commercial or financial relationships that could be construed as a potential conflict of interest.

## Publisher’s Note

All claims expressed in this article are solely those of the authors and do not necessarily represent those of their affiliated organizations, or those of the publisher, the editors and the reviewers. Any product that may be evaluated in this article, or claim that may be made by its manufacturer, is not guaranteed or endorsed by the publisher.
